# Detection of *Rickettsia* spp in Ticks by MALDI-TOF MS

**DOI:** 10.1371/journal.pntd.0003473

**Published:** 2015-02-06

**Authors:** Amina Yssouf, Lionel Almeras, Jérôme Terras, Cristina Socolovschi, Didier Raoult, Philippe Parola

**Affiliations:** 1 Aix Marseille Université, Unité de Recherche en Maladies Infectieuses et Tropicales Emergentes (URMITE), UM63, CNRS 7278, IRD 198 (Dakar, Sénégal), Inserm 1095, WHO Collaborative Center for Rickettsioses and Other Arthropod-Borne Bacterial Diseases, Faculté de Médecine, Marseille, France; 2 Hôpital Saint Joseph 26, Marseille, France; University of Texas Medical Branch, UNITED STATES

## Abstract

**Background:**

Matrix Assisted Laser Desorption/Ionization Time-of-Flight Mass Spectrometry (MALDI-TOF MS) has been shown to be an effective tool for the rapid identification of arthropods, including tick vectors of human diseases.

**Methodology/Principal Findings:**

The objective of the present study was to evaluate the use of MALDI-TOF MS to identify tick species, and to determine the presence of rickettsia pathogens in the infected Ticks. *Rhipicephalus sanguineus* and *Dermacentor marginatus* Ticks infected or not by *R. conorii conorii* or *R. slovaca*, respectively, were used as experimental models. The MS profiles generated from protein extracts prepared from tick legs exhibited mass peaks that distinguished the infected and uninfected Ticks, and successfully discriminated the *Rickettsia* spp. A blind test was performed using Ticks that were laboratory-reared, collected in the field or removed from patients and infected or not by *Rickettsia* spp. A query against our in-lab arthropod MS reference database revealed that the species and infection status of all Ticks were correctly identified at the species and infection status levels.

**Conclusions/Significance:**

Taken together, the present work demonstrates the utility of MALDI-TOF MS for a dual identification of tick species and intracellular bacteria. Therefore, MALDI-TOF MS is a relevant tool for the accurate detection of *Rickettsia* spp in Ticks for both field monitoring and entomological diagnosis. The present work offers new perspectives for the monitoring of other vector borne diseases that present public health concerns.

## Author Summary

Tick-borne rickettsioses include mild to life-threatening diseases in humans worldwide. When removing an attached tick from the human body, patients and physicians may have two questions: 1) is the tick a known vector of a human infectious disease, and 2) is the tick infected by a pathogenic agent that could have been transmitted during the attachment period? The morphological identification of Ticks is difficult, and requires expertise and specific documentation. The use of Matrix Assisted Laser Desorption/Ionization Time-of-Flight Mass Spectrometry (MALDI-TOF MS) has recently emerged as an effective, rapid and inexpensive tool to identify arthropods including Ticks. Here, we show the utility of MALDI-TOF MS for the dual identification of tick species and the rapid detection of *Rickettsia* spp in Ticks. Such results can be used to guide decisions related to specific patient monitoring or the administration of preventive treatment. Additionally, the low consumable costs, the minimum time required for sample preparation and the rapid availability of the results of MALDI-TOF MS could be useful for epidemiological studies and tick-borne disease monitoring via the dual identification of vectors and the pathogens they carry in one step. These results present new opportunities for the management of other vector-borne diseases that are of importance to public health.

## Introduction

Ticks are obligate hematophagous arthropods that parasitize vertebrates in almost all regions of the world and are currently considered to be the second-most important vectors of human infectious diseases worldwide, after mosquitoes [1]. Tick-borne rickettsioses are caused by obligate intracellular bacteria belonging to the spotted fever group of the genus *Rickettsia*. These zoonoses are among the oldest known vector-borne diseases, and include Mediterranean spotted fever, which is caused by *Rickettsia conorii conorii* and transmitted by the brown dog tick *Rhipicephalus sanguineus*. Additionally they include most of the emerging tick-borne diseases such as the infection caused by *R. slovaca* which is transmitted by *Dermacentor* spp [1, 2].

When removing an attached tick from the human body, patients and physicians may have two questions: 1) is the tick a known vector of human infectious disease, and 2) is the tick infected by a pathogenic agent? Identifying the species of the tick may alert the physician to the diseases that may appear, and knowledge of the infectious status of the tick is a key to evaluating the risk of disease transmission. Both pieces of information, if obtained quickly may be clinically helpful, particularly with regard to decisions about the use of antibiotic prophylactic treatment to prevent tick-borne diseases.

The routine method of identifying Ticks has traditionally been morphological identification using taxonomic keys, entomological expertise and specific documentation [1]. In the past decade, molecular tools have been developed to identify Ticks but these techniques also have their limitations including the selection of ideal primers, the requirement for technically time-consuming and expensive of PCR assays, and the availability of gene sequences in GenBank [1, 3]. More recently, we implemented the use of Matrix Assisted Laser Desorption/Ionization Time-of-Flight Mass Spectrometry (MALDI-TOF MS) in our laboratory as an effective tool to rapidly identify arthropods including Ticks [4–7]. Furthermore, with the creation of a database of reference spectra MALDI-TOF MS profiling of tick leg protein extracts will allow the rapid, cost-effective and accurate identification of Ticks.

For the detection and identification of *Rickettsia* species in infected Ticks, the most widely available tools remain molecular methods [1], and several *Rickettsia* DNA sequences can be detected and precisely identified in Ticks by different PCR methods [1]. However, to date, no system allows for the rapid and accurate identification of both the tick species and the *Rickettsia* spp that the Ticks harbor. Although the MALDI-TOF MS approach has emerged as a routine method for the identification and classification of bacteria for clinical diagnostics [8], no reference spectrum is available for the identification of intra-cellular *Rickettsia* in the commercial reference spectra database.

The aim of the present study was to determine whether it is possible, to simultaneously identify the tick species and the presence of an associated intra-cellular pathogen in a single assay. To test this, *Rh. sanguineus* and *D. marginatus* Ticks that were infected or not, by *R. c. conorii* or *R. slovaca*, respectively, were used as experimental models.

## Materials and Methods

### Ticks

Adult laboratory-reared *Rh. sanguineus* (n = 15) and *D. marginatus* (n = 20) were used, including rickettsia free specimens and specimens infected by *R. c. conorii* and *R. slovaca* respectively. *Rh sanguineus* were collected in France and Algeria and maintained at the URMITE laboratory. The *Rh. sanguineus* infected by *R. c. conorii* were obtained from specimens collected in the field, which were initially infected naturally by *R. c. conorii*. The vertical transmission of the *Rickettsia* in these Ticks during their laboratory rearing maintained the presence of *R. c. conorii* in this colony from generations to generation [9]. The presence of *R. c. conorii* was regularly confirmed by molecular biological analyses. The laboratory specimens were reared in an environmental incubator (19°C for *D. marginatus* and 25°C for *Rh sanguineus* with a relative humidity of 80–90%) and successive generations were obtained by allowing the Ticks to feed on rabbits as previously described [10]. The Ticks infected by *Rickettsia* spp were maintained in a biosafety level 3 laboratory (BSL-3). *D. marginatus* Ticks were also collected on dead wild boars killed by hunters in Southern France in order to obtain specimens infected by *R. slovaca* (see below). They *D. marginatus* Ticks were morphologically characterized using standard taxonomic keys [11]. For further analysis, each specimen was placed in 1.5 mL microcentrifuge tubes and immobilized or anesthetized at -20°C for 30 min. Whole Ticks were rinsed once with 70% ethanol for 2 min followed by 2 washes with distillated water. After air-drying, all of the legs were removed and two- to four-legs were used either for DNA extraction or sample preparation for MALDI-TOF MS analysis. Additionally, infected Ticks removed from patients including 2 specimens of *Rh. sanguineus* infected with *R. c. conorii*, 1 specimen of *Rh. sanguineus* infected with *R. massiliae* and 1 specimen of *D. marginatus* infected with *R. slovaca* were used. The presence of *Rickettsia* spp was previously confirmed by qPCR [4].

### *Rickettsia* culture and purification

All processing of infectious *Rickettsia* spp was carried out in a BSL 3 laboratory. *R. c. conorii* (ATCC N° VR613) and *R. slovaca* (CSUR N° R154) were grown into the cell line L929 (ATCC N° CCL-1) for approximately 7 days (+/- 2 days) at 32°C as previously described [12].]. To purify each *Rickettsia* strain, the infected L929 cells were centrifuged at 11650x g for 10 min. The pellets were rinsed twice in 30 mL of phosphate-buffered saline (PBS) (BIOMERIEUX/France) and centrifuged again at 11650x g for 10 min. The pellets were harvested in 18 mL of sterile PBS, vortexed, diluted in 12 mL of 2.5% concentrated Trypsin (Gibco®) and incubated at 37°C for 60 min. The suspensions were vortexed every 15mn and centrifuged at 11650x g for 10 min. This washing step was repeated three times using sterile PBS; the final suspensions were centrifuged and the pellets were collected in 1 mL of PBS. To eliminate the last cellular debris, two filtrations were performed using 5 μm and 0.8 μm filters (Millipore/France). The purity level and the quantification of the Rickettsia strains was evaluated by Gimenez staining [13] to detect residual cellular debris and to determine bacteria concentration. After purification, serial dilutions of each purified strain was performed in PBS and 10μL of each Rickettsia sample was applied to a 18 Well microscope slide (THERMO Cel-Line Diagnostic 6mm well), fixed by heat during 15min at 100°C, and stained by the Gimenez method [13]. Whole cells or cell debris were stained green and bacteria stained red. The purification rate was determined visually based on the absence of green labelling and the presence of red staining reflecting the individual purified bacteria. Bacteria concentration was estimated by counting all the bacteria in 5 different fields by well at two dilutions under microscopy.

After purification Rickettsia counting was also performed using flow cytometry (BD Accuri C6). The combination of side scatter (SSC) and forward (FSC) correlates with the cell size and the density of the particles of the sample analyzed. In this manner, a bacterial population can be distinguished according to the differences of its size and density without any fluorescent staining. In addition, flow cytometry allowed us to control for the purity of the bacterial based on the absence of whole cells or cell debris.

Serial dilutions of each purified Rickettsia bacteria strains in PBS buffer were performed to determine the optimal concentration for MALDI-TOF MS analysis. The rickettsial strain suspensions were then either immediately used for MALDI-TOF MS analysis or stored overnight at 4°C before MS analysis.

### DNA extraction and PCR detection of *Rickettsia*

DNA extractions were performed with one or two legs of each tick specimen included in the present study (laboratory and field specimens) using the EZ1 DNA Tissue kit (Qiagen, Hilden, Germany). Rickettsial DNA detection was performed by quantitative PCR using a CFX 96 Real Time System (BIO-RAD, Singapore) and the Eurogentec MasterMix Probe PCR kit (Qiagen, Hilden, Germany) following the manufacturer’s instructions. The presence of *R. c. conorii* and *R. slovaca* was determined using the primers R_conorii_6967 and R.slo_7128-R, respectively, which target tRNA intergenic spacers as previously described [14, 15]. A negative control (sterile water containing DNA extracted from uninfected Ticks maintained in laboratory colonies) and a positive control using DNA from *R. c. conorii* or *R. slovaca* strains were included in each respective test.

### Preparation of samples for MALDI-TOF MS analysis

**Ticks**. Two to four legs of *Rickettsia*-infected and uninfected Ticks were homogenized manually in 40 μL of 70% formic acid (Sigma, Lyon, France) and 40 μL of 100% acetonitrile (VWR Prolabo) using pellet pestles (Fischer Scientific). All homogenates were centrifuged at 6700 x g for 20 sec and 1 μL of each supernatant was spotted onto a steel target plate (Bruker Daltonics) in quadruplicate. Then, 1 μL of matrix suspension composed of saturated α-Cyano-4-hydroxy-cinnamic acid (CHCA) (Sigma), 50% acetonitrile, 10% trifluoroacetic acid (Sigma) and HPLC water was directly spotted onto each sample on the target plate. Following the drying of the matrix at room temperature, the target plate was immediately introduced into the MALDI-TOF MS instrument for analysis.

***Rickettsia* species**. For protein extraction from each *Rickettsia* species, a suspension of 500 μL of purified bacteria was centrifuged for 5 min at 14,000 x g. The supernatant was discarded and the pellet was washed twice in 500 μL of pure water, vortexed and centrifuged for 5 min at 14,000 x g. The pellet was then homogenized with 7.5 μL of 70% formic acid and 7.5 μL acetonitrile; after centrifugation at 14,000 x g for 5 min, 1 μL of supernatant was deposited on the target plate in quadruplicate and overlaid with 1 μL of CHCA matrix buffer.

**L929 cell line**. Uninfected cells were treated with 0.05% trypsin (1X), counted with Kova-Slide and washed twice in 10 mL of PBS; the cells were then centrifuged for 10 min at 262 x g. The pellet was homogenized in 1 mL of buffer to obtain a final concentration of 10^7^cells/mL. After a centrifugation at 14,000 x g for 5 min, 1 μL of the supernatant was deposited on the target plate in quadruplicate and overlaid with 1 μL of CHCA matrix buffer, as described above. The mass spectrometer was calibrated using the Bruker Bacterial Test Standard in the mass range of 2–20 kDa.

### Analysis of MS profiles

Protein mass profiles were acquired using a Microflex LT spectrometer (Bruker Daltonics) with Flex Control software (Bruker Daltonics). The spectra were recorded in a linear, positive ion mode with an acceleration voltage of 20 kV, within a mass range of 2,000–20,000 Da. Each spectrum corresponds to an accumulation of 240 laser shots from the same spot in six different positions. To control the loading on the steel target, the matrix quality and the MALDI-TOF apparatus performance, the matrix solution was loaded in duplicate onto each MALDI-TOF plate with or without Bacterial Test Standard (Bruker Protein Calibration Standard I). The spectrum profiles obtained were visualized with Flex analysis v.3.3 software and exported to ClinProTools version v.2.2 and MALDI-Biotyper v.3.0 (Bruker Daltonics, Germany).

### Comparisons of the mass spectra of tick specimens infected or not by Rickettsia spp

MALDI-TOF MS spectra from the leg protein extracts of 9 *D. marginatus* infected or not by *R. slovaca*, and 10 *Rh. sanguineus* infected or not by *R. c. conorii* were imported into ClinProTools v.2.2 (Bruker Daltonics, Germany) to identify the specific peaks related to the infection status of the tick. The parameters for ClinProTools software analysis were similar to those previously described [4]. An average spectrum was generated for each condition (*i.e*., tick species infected or not by *Rickettsia* spp), using the algorithm “average peak list calculation” tool within the range of 2–20 kDa. The detection of discriminating peak masses was performed by comparison of the average spectrum generated between two classes. The Genetic Algorithm (GA) model of the ClinProTools software was then used to automatically display a list of discriminating peak masses. Based on the selected peak masses, the values of Recognition Capability (RC) and Cross Validation (CV) were determined [16, 17]. The presence or absence of each discriminating peak masses generated by the model was verified by the comparison of each peak mass contained in the peak report created for each species, with the total average spectrum created from all the replicates between two classes (*i.e*., *Rickettsia*-infected and uninfected) for each tick species. Additionally the peak mass lists of each *Rickettsia* strain were retrieved from the Flex analysis v.3.3 software.

### Blind tests

The accuracy of MALDI-TOF MS for the detection both of the Ticks and pathogens was assessed in a validation step involving a blind test using other tick specimens that were infected or not by *Rickettsia* spp, including Ticks collected in the field or removed from patients. MALDI-TOF MS spectra from the leg protein extracts of 3 uninfected *D. marginatus*, 3 *D. marginatus* infected by *R. slovaca*, 2 uninfected *Rh. sanguineus* and 4 *Rh. sanguineus* infected with *R. c. conorii*, were used for a blind test (Blind test 1) with 1 to 4 new specimens per species against our laboratory’s database of reference spectra for (Database 1). This database includes the leg protein spectra of 6 rickettsia free tick species (*Amblyomma variegatum* infected by *R. africae*, *Rh. sanguineus*, *Hyalomma marginatum rufipes*, *Ixodes ricinus*, *D. marginatus* and *D. reticulatus*), 30 mosquito species (*Anopheles gambiae* molecular form M and *An. gambiae* molecular form S, *An. funestus, An. ziemanni, An. arabiensis, An. wellcomei, An. rufipes, An. pharoensis, An. coustani, An. claviger, An. hyrcanus, An. maculipennis*, C*ulex quinquefasciatus*, *Cx. pipiens*, *Cx. modestus, Cx. insignis, Cx. neavei, Ae. albopictus*, *Aedes excrucians, Ae vexans, Ae. rusticus, Ae. dufouri, Ae. cinereus, Ae. fowleri, Ae. aegypti*, *Ae. caspius*, *Mansonia uniformis, Orthopodomyia reunionensis, Coquillettidia richiardii* and *Lutzia tigripes*,), and other arthropods including louse (*Pediculus humanus corporis*), triatomine (*Triatoma infestans*) and bedbugs (*Cimex lectularius*), as well as the spectra obtained from the bodies (without the abdomens) of 5 flea species (*Ctenocephalides felis*, *Ct. canis, Archaeopsylla erinacei, Xenopsylla cheopis* and *Stenoponia tripectinata*) [4–7]. Then, MALDI-TOF MS spectra from uninfected *D. marginatus* (n = 4), *D. marginatus* infected by *R. slovaca* (n = 4), uninfected *Rh. sanguineus* (n = 4) and *Rh. sanguineus* infected with *R. c. conorii* (n = 5) were added to our database; this upgraded database is referred to as Database 2. The same specimens of *D. marginatus*, *D. marginatus* infected by *R. slovaca*, uninfected *Rh. sanguineus* and *Rh. sanguineus* infected with *R. c. conorii*, were tested in a blind test against Database 2 (Blind test 2). Additionally, the spectra from the leg protein extracts of 3 Ticks removed from 3 patients were also tested against Database 2. The presence of *Rickettsia* spp was previously confirmed by qPCR including 1 specimen of *Rh. sanguineus* infected with *R. c. conorii* (Ct = 22), 1 specimen of *Rh. sanguineus* infected with *R. massiliae* (Ct = 24), and 1 specimen of *D. marginatus* infected with *R. slovaca* (Ct = 19) (Table 1) [4].

**Table 1:** Tick species selected for blind tests against the arthropod MALDI-TOF MS reference databases.

**Species**	**Source**	**Detection of *Rickettsia* spp by specific qPCR (Cycle Threshold)**	**Identification and higher LSVs against Database 1^a^**	**Identification and higher LSVs against Database 2^b^**
*D. marginatus*	Laboratory	(-)*	*D. marginatus* (2.431)	*D. marginatus* (2.431)
*D. marginatus*	Laboratory	(-)*	*D. marginatus* (2.298)	*D. marginatus* (2.298)
*D. marginatus*	Laboratory	(-)*	*D. marginatus* (2.449)	*D. marginatus* (2.449)
*D. marginatus* infected by *R. slovaca*	Laboratory	19.91	*D. marginatus* (1.817)	*D. marginatus* infected by *R. slovaca* (1.864)
*D. marginatus* infected by *R. slovaca*	Laboratory	17.7	*D. marginatus* (1.756)	*D. marginatus* infected by *R. slovaca* (2.193)
*D. marginatus* infected by *R. slovaca*	Laboratory	21.1	*D. marginatus* (1.831)	*D. marginatus* infected by *R. slovaca* (1.941)
*D. marginatus* infected by *R. slovaca*	Removed from patient	19	*D. marginatus* (1.793)	*D. marginatus* infected by *R. slovaca* (1.857)
*Rh. sanguineus/*	Laboratory	(-)*	*Rh. sanguineus* (2.277)	*Rh. sanguineus* (2.277)
*Rh. sanguineus*	Laboratory	(-)*	*Rh. sanguineus* (2.305)	*Rh. sanguineus* (2.305)
*Rh. sanguineus* infected by *R. c. conorii*	Laboratory	26.69	*Rh. sanguineus* (2.101)	*Rh. sanguineus* infected by *R. c. conorii* (2.243)
*Rh. sanguineus* infected by *R. c. conorii*	Laboratory	26.46	*Rh. sanguineus* (1.845)	*Rh. sanguineus* infected by *R. c. conorii* (2.406)
*Rh. sanguineus* infected by *R. c. conorii*	Laboratory	26.18	*Rh. sanguineus* (1.92)	*Rh. sanguineus* infected by *R. c. conorii* (2.242)
*Rh. sanguineus* infected by *R. c. conorii*	Laboratory	30.09	*Rh. sanguineus* (1.98)	*Rh. sanguineus* infected by *R. c. conorii* (2.047)
*Rh. sanguineus* infected by *R. c. conorii*	Removed from patient	22	*Rh. sanguineus* (2.119)	*Rh. sanguineus* infected by *R. c. conorii* (2.216)
*Rh. sanguineus* infected by *R. massiliae*	Removed from patient	24	*Rh. sanguineus* (2.057)	*Rh. sanguineus* (2.057)

* Negative qPCR;

^a^ Database 1 is composed of 6 tick, 30 mosquito and 5 flea species and other arthropods such as a louse (*Pediculus humanus corporis*), triatomines (*Triatoma infestans*) and bedbugs (*Cimex lectularius*);

^b^ Database 2 is composed of Database 1 plus *Rickettsia* spp infected by *Rh. sanguineus* and *D. marginatus*; LSVs, log score values.

The reliability of the identification was estimated based on the Log Score values (LSVs) exhibited by the MALDI-Biotyper software, between 0 and 3. These LSVs correspond to the degree of homology between the query mass spectra and the reference spectra. An LSV was obtained for each spectrum of the samples tested.

### Ethical statement

The maintenance of laboratory colony of *Rhipicephalus sanguineus* and *Dermacentor marginatus* Ticks [18] has been approved by the Institutional Animal Care and Use Committee of the Faculty of Medicine at Aix-Marseille University, France. The collection of *Dermacentor marginatus* Ticks in the field did not involve privately owned, wildlife, national park or other protected areas and endangered or protected species.

## Results

### Confirmation of rickettsial infection in Ticks

When the legs of 15 *Rh. sanguineus* specimens including 8 specimens presumably infected with *R. c. conorii* and 7 *Rickettsia*-free specimens from the laboratory colony were tested by qPCR, *R. c. conorii* DNA was detected in 100% (8/8) of the *Rh sanguineus* legs predicted to be infected by this bacterium, with a mean Ct ± SD value of 28.76 ±3.27 (Table 1). As expected, *R. c. conorii* DNA was not detectable in the *Rh. sanguineus Rickettsia*-free specimens. When the legs of 12 *D. marginatus* collected in the field were tested by qPCR, 58% (7/12) of the tick legs tested positive for the presence of *R. slovaca* with a mean Ct ± SD value of 23.93 ± 5.62 (Table 1). Additionally, the absence of *R. slovaca* from the laboratory reared *D. marginatus* colony was confirmed by quantitative PCR.

### *Rickettsia* culture and purification

Gimenez straining was performed to determine the purity and concentration of each Rickettsia strain (S1A and S1B Fig.). The absence of green labelling indicated that the purified bacteria samples were free of cells and cell debris. The purity of the samples was confirmed by flow cytometry (BD ACCURI C6 instrument) to detect a homogeneous population of bacteria. Serial dilution of the purified bacteria samples was performed to determine the Rickettsia concentration. Flow cytometry and direct counting on slides by Gimenez labelling led to similar results (S1C and S1D Fig.). The concentration of each purified strain was of 1.6 x10^7^ bacteria /mL and 1.35 × 10^7^ bacteria /mL for *R. c. conorii* and for *R. slovaca*, respectively (S1E Fig.) for the MALDI-TOF MS analysis.

### MALDI-TOF MS spectra

**Fig 1:**
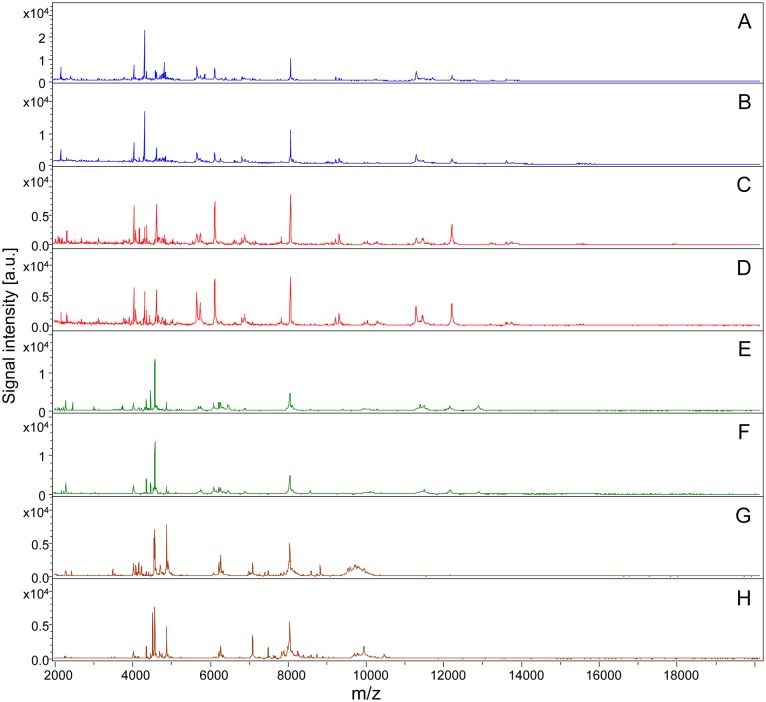
Comparison of MALDI-TOF MS profiles of Ticks infected or not by *Rickettsia* spp. Representative spectra from biological replicates of *Rh. sanguineus* (A, B), *Rh. sanguineus* infected by *R. conorii conorii* (C, D), *D. marginatus* (E, F) and *D. marginatus* infected by *R. slovaca* (G, H) were aligned using Flex analysis 3.3 software. a.u., arbitrary units; m/z, mass-to-charge ratio.

**Fig 2:**
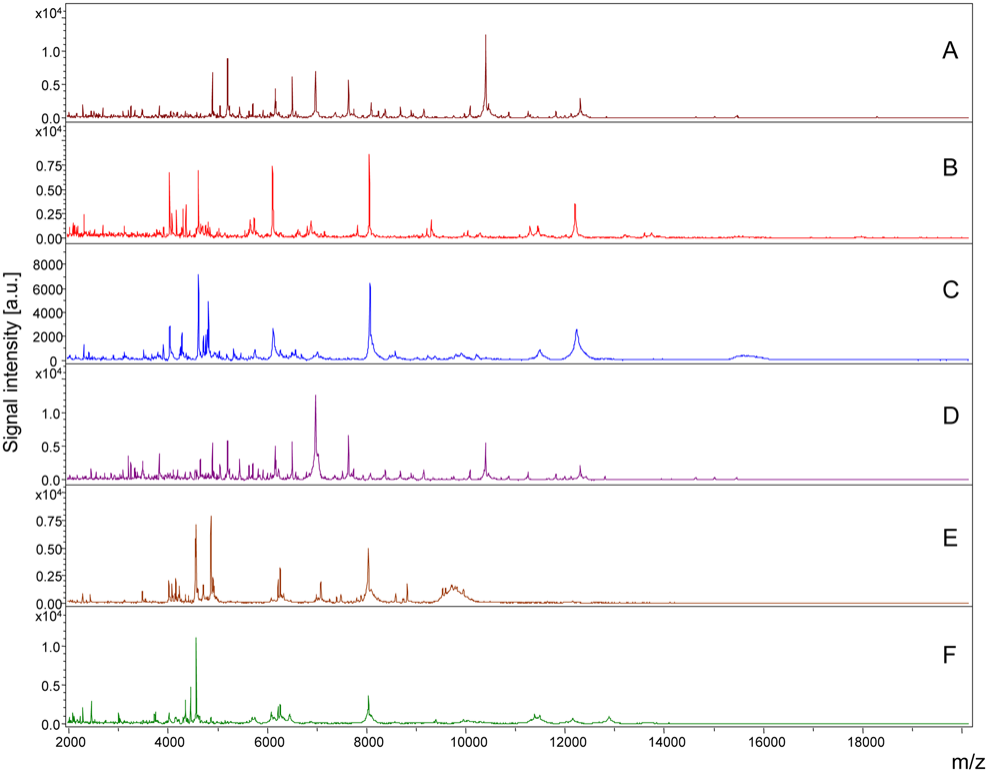
Alignment of MALDI-TOF MS profiles of *Rickettsia* strains and tick species infected or not by *Rickettsia* using Flex analysis 3.3 software. Representative spectra of a purified *R. conorii conorii* strain (A), *Rh. sanguineus* (B), *Rh. sanguineus* infected by *R. conorii conorii* (C), a purified *R. slovaca* strain (D), *D. marginatus* (E) and *D. marginatus* infected by *R. slovaca* (F) are presented. a.u., arbitrary units; m/z, mass-to-charge ratio.

Legs from a total of 19 *Rickettsia*-infected and 13 uninfected specimens belonging to *Rh. sanguineus* (n = 17) and *D. marginatus* (n = 15) were subjected to MALDI-TOF MS analysis (Table 1). Although one leg of adult tick was sufficient to generate an accurate MS spectra, to increase the rate of identification, at least two adult tick legs should be included in the preparation for mass spectra analyses (Yssouf et al 2013). Similar MALDI-TOF MS spectra profiles from the leg protein extracts were obtained for each tick species and infectious status. Representative MS profiles with high intensities peaks in the range of 2–20 kDa are presented in Fig. 1. Using Flex analysis software, the alignment of the leg MALDI-TOF MS spectra of 2 uninfected specimens of *R. sanguineus* and 2 specimens of *Rh. sanguineus* infected by *R. c. conorii*, confirmed the reproducibility of the spectra and also revealed changes in the MS pattern according to the infectious status. Comparable results were obtained from MS spectra of *D. marginatus* specimens infected or not by *R. slovaca*. Although several protein peaks were conserved in the spectra from specimens belonging to the same species, modifications of the MS patterns were detectable in *Rickettsia*-infected specimens compared to uninfected specimens (Fig. 2). Technical and biological replicates yielded reproducible spectra (Fig. 1). The spectra of at least 4 specimens of each species (infected and uninfected) were added to our arthropod database (Database 1) in MALDI-Biotyper 3.0, which was designated as Database 2. In parallel, MALDI-TOF MS spectra of each *Rickettsia* strains were compared to that of the L929 cell line. The alignment of the spectrum profiles of the strains with the cell line using Flex analysis software revealed the absence of peaks with identical masse-to-charge ratios, supporting the conclusion that Rickettsia strains were not contaminated by L929 cell proteins and that the MS spectra corresponded to the Rickettsia strains.

### Singularity of the MS patterns according to tick species and infectious status

**Fig 3:**
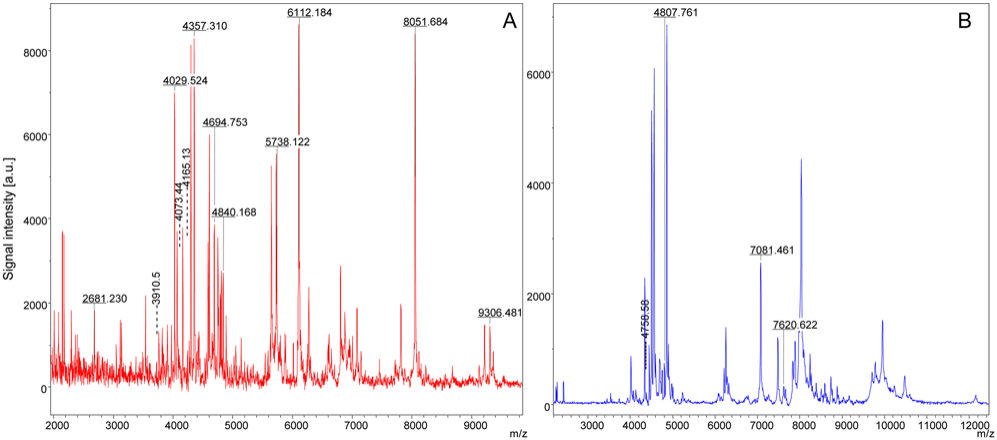
Location of discriminating peak masses shared between the spectra acquired from *Rickettsia*-infected specimens and the corresponding *Rickettsia* strain using Flex analysis software 3.3. The alignment spectra comparing the infected specimen and the corresponding strain spectra are shown in detail. (A) *R. conorii conorii* shared discriminating peak masses located on the MS profile of *Rh. sanguineus* infected by *R. conorii conorii*. (B) *R. slovaca* shared discriminating peak masses located on the MS profile of *D. marginatus* infected by *R. slovaca*.

To determine whether the mass spectra data were suitable for the identification of discriminating peaks (m/z-values) according to the *Rickettsia*-infectious status, 16 to 20 MS spectra per group were selected for further analysis and loaded into the ClinProTools software. Among the *Rh. sanguineus* and *D. marginatus* Ticks that were infected or not, by *R. c. conorii* or *R. slovaca*, respectively, 76 spectra from 19 specimens that were selected for the MALDI-Biotyper database were imported into the ClinProTools software. The Genetic Algorithm model displayed the peak masses that discriminate between the Ticks that were infected or not by *Rickettsia* spp with RC and CV values of 100% for both comparisons. After verification of the peak report in the averaged spectrum of the *Rh. sanguineus* species, 30 biomarker masses were identified that could distinguish *Rh. sanguineus* specimens that were infected or not by *R. c. conorii* (Table 2). Among them, 22 peak masses were observed uniquely in the *R. conorii*-infected specimens and 8 peak masses were associated with the uninfected *Rh. sanguineus* specimens (Table 2). To confirm the specificity of several of these discriminant biomarker masses, a comparison of the MSP between *Rh. sanguineus* infected by *R. c. conorii* and the purified *R. c. conorii* strain was performed (Table 2). Twelve peak masses were common to both samples, and they were localized in the spectra of *Rh. sanguineus* infected by *R. c. conorii* using Flex analysis software (Fig. 3A). Using a comparable strategy for *D. marginatus* specimens, 35 discriminating peak masses were identified, among which 21 peak masses were specific to spectra from *D. marginatus* infected by *R. slovaca* (Table 3). Moreover, among these 21 specific peak masses, 4 were shared between *D. marginatus* infected by *R. slovaca* and the purified *R. slovaca* strain. These 4 peak masses were localized on the spectra profiles of infected *D. marginatus* using the Flex analysis software (Fig. 3B).

**Table 2:** Peak masses distinguishing uninfected and *R. c conorii*-infected *Rh. sanguineus* Ticks and the determination of the peak masses shared with a purified *R. c. conorii* strain based on statistical analysis with ClinProTools.

**Mass (Da)**	***Rh. sanguineus* non infected**	***Rh. sanguineus* infected by *R.c. conorii***	**Strain of *R.c. conorii***
2148.78	No	Yes	No
2177.18	No	Yes	No
2279.34	Yes	No	No
2304.11	No	Yes	No
2586.36	Yes	No	No
2686.07	No	Yes	Yes
3121.32	No	Yes	No
3910.5	No	Yes	Yes
4020.51	Yes	No	No
4030.21	No	Yes	Yes
4073.44	No	Yes	Yes
4165.13	No	Yes	Yes
4350.22	No	Yes	Yes
4358.24	No	Yes	No
4456.14	Yes	No	No
4584.2	No	Yes	Yes
4841.34	No	Yes	Yes
4868.62	Yes	No	No
5730.84	No	Yes	Yes
5741.13	No	Yes	No
6082.26	Yes	No	No
6108.64	No	Yes	Yes
6220.67	Yes	No	No
6811.25	No	Yes	No
6881.24	No	Yes	No
7817.31	No	Yes	No
8042.45	Yes	No	No
8056.12	No	Yes	Yes
9307.75	No	Yes	Yes
12207.38	No	Yes	No
**Total**	**8**	**22**	**12**

**Table 3:** Peak masses distinguishing uninfected and *R.slovaca*-infected D. marginatus Ticks and the determination of the peak masses shared with a purified R. slovaca strain based on statistical analysis with ClinProTools.

**Mass (Da)**	***D.marginatus* uninfected**	***D.marginatus* infected by *R. slovaca***	**Strain of *R. slovaca***
2043.33	Yes	No	No
2082.79	Yes	No	No
2585.65	Yes	No	No
3493.53	Yes	No	No
3542.88	Yes	No	No
3587.51	Yes	No	No
3960	Yes	No	No
4136.53	No	Yes	No
4173.02	Yes	No	No
4226.84	Yes	No	No
4305.29	Yes	No	No
4510.78	No	Yes	No
4592.4	Yes	No	No
4629.69	Yes	No	No
4694.34	No	Yes	No
4758.58	No	Yes	Yes
4808.98	No	Yes	Yes
4906.81	No	Yes	No
4923.94	Yes	No	No
6336.71	No	Yes	No
7082.05	No	Yes	Yes
7485.1	No	Yes	No
7626	No	Yes	Yes
7665	No	Yes	No
7835.64	No	Yes	No
7894.93	No	Yes	No
8241.9	No	Yes	No
8389.5	No	Yes	No
8596.3	No	Yes	No
8738.5	No	Yes	No
8846.1	No	Yes	No
9785	No	Yes	No
9957.33	No	Yes	No
10483.7	No	Yes	No
11316.91	Yes	No	No
**Total**	**14**	**21**	**4**

### Blind tests

A total 15 specimens, including uninfected and *Rickettsia*-infected Ticks, were queried successively against the MS reference Database 1 and Database 2 (*i.e*., Database 2 = Database 1 plus the spectra from *Rickettsia*-infected Ticks). Using Database 1, the blind test yielded 100% correct identification at the species level for the specimens tested irrespective of their infectious status and their origin of collection (*i.e*., Ticks that were laboratory-reared, collected in the field or removed from patients). The LSVs of the first top-ranking hits against Database 1 varied from 1.756 to 2.449 (Table 1). Interestingly, the tick specimens infected by *Rickettsia* spp had lower LSVs than the uninfected specimens. The same specimens were then tested against Database 2, and 100% of the specimens tested possessing a corresponding reference spectrum in Database 2 were correctly identified at the levels of tick species and infectious status (Table 1). Moreover, with the exception of the *Rh. sanguineus* specimen infected by *R. massiliae*, only the LSVs from *Rickettsia*-infected Ticks were increased, and all of these specimens had an LSV larger than 1.85. Interestingly, no association was observed between the cycle threshold value of qPCR and the LSVs. Although no reference spectrum was included in the database for the *Rh. sanguineus* specimen infected by *R. massiliae*, it was correctly identified at the level of the tick species as an uninfected *Rh. sanguineus* specimen, with an LSV greater than 2.

## Discussion

After the demonstration that MALDI-TOF MS profiling is an accurate tool to identify arthropods [19–23], including vectors of infectious diseases such as Ticks [4, 24], the possibility of identifying the presence of microorganisms inside the vectors became evident.

Recently, we showed that the MALDI-TOF MS approach could successfully detect and screen *Borrelia spp* in their soft tick vectors [25]; the legs of Ticks were used for the dual identification of tick species and the detection of Borrelia relapsing fever [25]. It has also been shown that MALDI-TOF-MS could be employed for the rapid screening of pathogens in tick vectors within the same experiment used for tick identification.

Here, we assess the application of MALDI-TOF MS for the detection of intracellular *Rickettsia* bacteria and the identification of their respective tick vectors. The present study revealed that the MALDI-TOF MS spectra obtained from two to four tick leg protein extracts were sufficient to accurately identify both the arthropod species and its infectious status. The advantage of performing both of these identifications using only legs is that allows the remaining body parts to be utilized for other analyses. In our study, the infection of Ticks by *Rickettsia* spp was confirmed by molecular approaches using DNA extracted from the remaining tick legs. In addition to validation of the tick infectious status, *Rickettsia* specific quantitative PCR confirmed the dissemination of these bacteria in the tick body including the legs.

To evaluate the consequences of *Rickettsia* infection on the MS profiles of Ticks, we compared the spectra produced by *Rh. sanguineus* and *D. marginatus* Ticks that were, infected or not by *R. c. conorii* and *R. slovaca*, respectively. The alignment of the MS profiles from *Rh. sanguineus* Ticks that were uninfected or infected by *R. c. conorii* led to incomplete superimposable protein profiles. Similar results were obtained for *D. marginatus* specimens that were infected or not by *R. slovaca*. The uniqueness of the MS profiles according to the tick species and infectious status suggests that the detected variations could be attributed to the presence of *Rickettsia* spp. The analysis of the spectra with ClinProTools revealed the existence of specific discriminating peak masses between infected and uninfected specimens. In total 30 and 35 biomarker mass sets distinguished the uninfected specimens of *Rickettsia* spp from the infected specimens of *D. marginatus* and *Rh. sanguineus* species, respectively. Interestingly, although the majority of the discriminating peaks appeared in the protein profiles of the infected Ticks, some were not maintained. This loss of some peak masses could be detrimental to the level of significant identification (*i.e*., LSVs) of Ticks at the species level. Effectively, our blind test experiments indicated that the LSVs of the infected specimens were lower than those of the uninfected Ticks when compared with Database 1, which included only uninfected specimens. In the future, it will be necessary to test the infectious status of a specimen of new species prior to including the results in the reference database. Moreover, the addition of MS spectra from specimens infected with pathogens will improve the identification of arthropod species and the pathogens that they carry.

In addition, among the discriminating peak mass sets found in the infected Ticks, few of them were shared with their respective purified *Rickettsia* strains. These masses could correspond to *Rickettsia*-specific proteins. Moreover, some discriminating peaks detected uniquely in the *Rickettsia*-infected Ticks were not present in the spectra peaks of the bacteria strains. These differential peak masses could be attributed either to *Rickettsia* strains (*i.e*., variations between laboratory and field strains) [26] or to a response of the Ticks to infection [27]. Complementary experiments are needed to test these hypotheses.

The validity of the databases was established by blind tests using infected and uninfected specimens. A query against Database 2 demonstrated that all the specimens possessing reference spectra in the database were correctly identified at the level of the tick species and the *Rickettsia*-infectious status. Moreover, 86% (n = 12/14) of these spectra presented LSVs greater than 1.9, which is considered to be reliable score for bacterial species identification [28]. Thus, the spectral variations that are detected following *Rickettsia* infection are sufficient to avoid cross-recognition between uninfected and infected Ticks. Moreover, the presence of *Rickettsia* in the Ticks did not mask the protein profiles for unambiguous identification at the species level (*e.g*., querying the MS spectra against Database 1). These results are in agreement with a previous study showing that these variations do not interfere with species determination [24]. However, the absence of corresponding reference spectra in Database 2 for *Rh. sanguineus* infected by *R. massiliae* resulted in an incorrect identification of this sample. It is necessary to complete this database with additional tick species infected by *Rickettsia* strains.

### Conclusions

The present study shows that MALDI-TOF MS can be used to reliably identify tick species infected or not by *Rickettsia* spp without the use of a molecular method requiring DNA sequence information. It is important to note that no *Rickettsia* spp spectrum is available in the Bruker reference database and that this is the first analysis of Rickettsia strain by MALDI-TOF MS. This work also demonstrated that MALDI-TOF MS could be applied for the rapid detection of *Rickettsia* spp in Ticks removed from patients. The rapid determination of a tick’s identity and it infectious status should guide decisions related to specific patient monitoring or the administration of preventive treatment. Additionally, the low consumable costs, minimal time required for sample preparation and rapid availability of the results of MALDI-TOF MS could be useful for epidemiological studies and the monitoring of tick-borne diseases via the dual identification of vectors and their borne pathogen in one step. The main obstacle to the use of the MALDI-TOF MS approach is the cost of acquiring the machine, but its use is cost effective thereafter [29]. These results also open new doors for the monitoring and management of other vector-borne diseases that are of importance for public health in human and veterinary medicine. For example, it would be advantageous to test whether MALDI-TOF MS, which has been shown to be a relevant tool for the identification of mosquito species [5, 7, 29, 30], could be useful for detecting the *Plasmodium*-infectious status of mosquito malaria vectors.

## Supporting Information

S1 Fig Purification and quantification of Rickettsia strains. A 100x magnification image of *Rickettsia slovaca* grown in L929 cells through an optical microscope before purification (A) and after purification (B). Representative FSC-A vs SSC-A plots of *R. conorii* (C) and. *R. slovaca* strains in logarithmic scale for counting bacteria. Purified Rickettsia strains were diluted in PBS at 10^-1^ and 10^-2^ and then analyzed on an ACCURI C6 (Medium fluidics speed and Threshold at 10000 for analysis of small particles). (E) Raw quantification data and calculated concentration of each *Rickettsia* strains are presented.
(TIF)

## References

[1] Parola P, Raoult D (2001) Ticks and tickborne bacterial diseases in humans: an emerging infectious threat. Clin Infect Dis 32: 897–928. 10.1086/319347 11247714

[2] Parola P, Paddock CD, Socolovschi C, Labruna MB, Mediannikov O, Kernif T, Fournier PE, Raoult D (2013) Update on Tick-Borne Rickettsioses around the World: a Geographic Approach. Clin Microbiol Rev 26: 657 10.1128/CMR.00032-13 24092850 PMC3811236

[3] Zhang RL, Zhang B (2014) Prospects of using DNA barcoding for species identification and evaluation of the accuracy of sequence databases for Ticks (Acari: Ixodida). Ticks and Tick-borne Diseases 5: 352–358. 10.1016/j.ttbdis.2014.01.001 24656809

[4] Yssouf A, Flaudrops C, Drali R, Kernif T, Socolovschi C, Berenger JM, Raoult D, Parola P (2013) Matrix-assisted laser desorption ionization-time of flight mass spectrometry for rapid identification of tick vectors. J Clin Microbiol 51: 522–528. 10.1128/JCM.02665-12 23224087 PMC3553915

[5] Yssouf A, Socolovschi C, Flaudrops C, Ndiath MO, Sougoufara S, Dehecq JS, Lacour G, Berenger JM, Sokhna CS, Raoult D, Parola P (2013) Matrix-assisted laser desorption ionization-time of flight mass spectrometry: an emerging tool for the rapid identification of mosquito vectors. PLoS One 8: e72380 10.1371/journal.pone.0072380 23977292 PMC3744494

[6] Yssouf A, Socolovschi C, Leulmi H, Kernif T, Bitam I, Audoly G, Almeras L, Raoult D, Parola P (2014) Identification of flea species using MALDI-TOF/MS. Comp Immunol Microbiol Infect Dis 37: 153–157. 10.1016/j.cimid.2014.05.002 24878069

[7] Yssouf A, Parola P, Lindstrom A, Lilja T, L’Ambert G, Bondesson U, Berenger JM, Raoult D, Almeras L (2014) Identification of European mosquito species by MALDI-TOF MS. Parasitol Res 113: 2375–2378. 24737398 10.1007/s00436-014-3876-y

[8] Seng P, Rolain JM, Fournier PE, La Scola B, Drancourt M, Raoult D (2010) MALDI-TOF-mass spectrometry applications in clinical microbiology. Future Microbiol 5: 1733–1754. 21133692 10.2217/fmb.10.127

[9] Socolovschi C, Bitam I, Raoult D, Parola P (2009) Transmission of *Rickettsia conorii conorii* in naturally infected *Rhipicephalus sanguineus* . Clin Microbiol Infect 15 Suppl 2: 319–321. 10.1111/j.1469-0691.2008.02257.x 19438619

[10] Vu Hai V, Almeras L, Audebert S, Pophillat M, Boulanger N, Parola P, Raoult D, Pages F (2013) Identification of salivary antigenic markers discriminating host exposition between two European Ticks: *Rhipicephalus sanguineus* and *Dermacentor reticulatus* . Comparative Immunology, Microbiology and Infectious Diseases 36: 39–53. 10.1016/j.cimid.2012.09.003 23040662

[11] A. Estrada Pena (2004) Ticks of domestic Animals in the Mediterranean Region.

[12] Masala G, Chisu V, Satta G, Socolovschi C, Raoult D, Parola P (2012) *Rickettsia slovaca* from *Dermacentor marginatus* Ticks in Sardinia, Italy. Ticks Tick Borne Dis 3: 393–395. 23140897 10.1016/j.ttbdis.2012.10.007

[13] Gimenez DF (1964) Staining Rickettsiae in yolksack cultures. Staining Technol 39: 135–140. 10.3109/10520296409061219 14157454

[14] Ogata H, Audic S, Barbe V, Artiguenave F, Fournier E, Raoult D, Claverie J (2000) Selfish DNA in protein-coding genes of Rickettsia. Science 290: 347–350. 10.1126/science.290.5490.347 11030655

[15] Fournier PE, El Karkouri K, Robert C, Medigue C, Raoult D (2012) Complete genome sequence of *Rickettsia slovaca*, the agent of tick-borne lymphadenitis. J Bacteriol 194: 1612 10.1128/JB.06625-11 22374949 PMC3294842

[16] Calderaro A, Gorrini C, Piccolo G, Montecchini S, Buttrini M, Rossi S, Piergianni M, Arcangeletti MC, De Conto F, Chezzi C, Medici MC (2014) Identification of *Borrelia* species after creation of an in-house MALDI-TOF MS database. PLoS One 9: e88895 10.1371/journal.pone.0088895 24533160 PMC3923052

[17] Calderaro A, Piccolo G, Gorrini C, Montecchini S, Buttrini M, Rossi S, Piergianni M, De CF, Arcangeletti MC, Chezzi C, Medici MC (2014) *Leptospira* species and serovars identified by MALDI-TOF mass spectrometry after database implementation. BMC Res Notes 7: 330 10.1186/1756-0500-7-330 24890024 PMC4048046

[18] Matsumoto K, Brouqui P, Raoult D, Parola P (2005) Experimental infection models of Ticks of the *Rhipicephalus sanguineus* group with *Rickettsia conorii* . Vector Borne Zoonotic Dis 5: 363–372. 16417432 10.1089/vbz.2005.5.363

[19] Campbell PM (2005) Species differentiation of insects and other multicellular organisms using matrix-assisted laser desorption/ ionization time of flight mass spectrometry protein profiling. systematic Entomology 30: 186–190. 10.1111/j.1365-3113.2004.00279.x

[20] Feltens R, Gorner R, Kalkhof S, Groger-Arndt H, Von BM (2010) Discrimination of different species from the genus *Drosophila* by intact protein profiling using matrix-assisted laser desorption ionization mass spectrometry. BMC Evol Biol 10: 95 10.1186/1471-2148-10-95 20374617 PMC2858148

[21] Kaufmann C, Schaffner F, Ziegler D, Pfluger V, Mathis A (2012) Identification of field-caught *Culicoides* biting midges using matrix-assisted laser desorption/ionization time of flight mass spectrometry. Parasitology 139: 248–258. 10.1017/S0031182011001764 22008297

[22] Kaufmann C, Ziegler D, Schaffner F, Carpenter S, Pfluger V, Mathis A (2011) Evaluation of matrix-assisted laser desorption/ionization time of flight mass spectrometry for characterization of *Culicoides nubeculosus* biting midges. Medical and Veterinary Entomology 25: 32–38. 21118284 10.1111/j.1365-2915.2010.00927.x

[23] Modika RP, Flores Vargas RD, Jones MGK (2005) Identification of aphid species using protein profiling and matrix-assisted laser desorption/ionisation time-of-flight mass spectrometry. Entomologia Experimentalis et Applicata 117: 243–247.

[24] Karger A, Kampen H, Bettin B, Dautel H, Ziller M, Hoffmann B, Suss J, Klaus C (2012) Species determination and characterization of developmental stages of Ticks by whole-animal matrix-assisted laser desorption/ionization mass spectrometry. Ticks Tick Borne Dis 3: 78–89. 10.1016/j.ttbdis.2011.11.002 22487425

[25] Fotso Fotso A, Mediannikov O, Diatta G, Flaudrops C, Parola P, Drancourt M (2014) MALDI-TOF mass spectrometry detection of relapsing fever *Borrelia crocidurae* in *Ornithodoros sonrai* Ticks. PLoS Negl Trop Dis, in press. 10.1371/journal.pntd.0002984 PMC4109908 25058611

[26] James A C, El-Hage N, Miller JC, Babb K, Stevenson B (2001) *Borrelia burgdorferi* RevA Antigen Is a Surface-Exposed Outer Membrane Protein Whose Expression Is Regulated in Response to Environmental Temperature and pH. Infect Immun 69: 5286. 11500397 10.1128/IAI.69.9.5286-5293.2001 PMC98637

[27] Antunes S, Galindo RC, Almazan C, Rudenko N, Golovchenko M, Grubhoffer L, Shkap V, do RV, de la Fuente J, Domingos A (2012) Functional genomics studies of *Rhipicephalus* (Boophilus) *annulatus* Ticks in response to infection with the cattle protozoan parasite, *Babesia bigemina* . Int J Parasitol 42: 187–195. 10.1016/j.ijpara.2011.12.003 22265898

[28] Fournier PE, Couderc C, Buffet S, Flaudrops C, Raoult D (2009) Rapid and cost-effective identification of *Bartonella* species using mass spectrometry. J Med Microbiol 58: 1154–1159. 10.1099/jmm.0.009647-0 19528172

[29] Müller P, Pflüger V, Wittwer M, Ziegler D, Chandre F, Simard F, Lengeler C (2013) Identification of cryptic *Anopheles* mosquito species by molecular protein profiling. PLoS One 8: e57486 10.1371/journal.pone.0057486 23469000 PMC3585343

[30] Schaffner F, Kaufmann C, Pfluger V, Mathis A (2014) Rapid protein profiling facilitates surveillance of invasive mosquito species. Parasit Vectors 7: 142 10.1186/1756-3305-7-142 24685094 PMC4022357

