# Supplementary figures and images for: Detection of Rickettsia spp in Ticks by MALDI-TOF MS
Source: PLoS Negl Trop Dis. 2015 Feb 6;9(2):e0003473. doi: 10.1371/journal.pntd.0003473 (PMC4319929; doi:10.1371/journal.pntd.0003473)

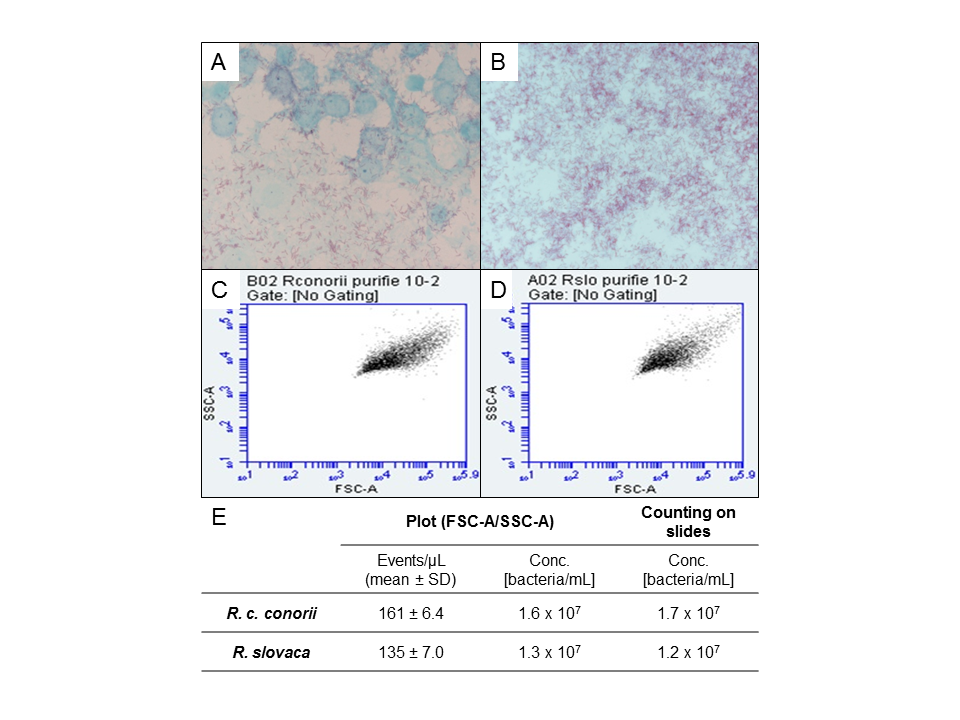

Supplement: S1 Fig — A 100x magnification image of Rickettsia slovaca grown in L929 cells through an optical microscope before purification (A) and after purification (B). Representative FSC-A vs SSC-A plots of R. conorii (C) and. R. slovaca strains in logarithmic scale for counting bacteria. Purified Rickettsia strains were diluted in PBS at 10-1 and 10-2 and then analyzed on an ACCURI C6 (Medium fluidics speed and Threshold at 10000 for analysis of small particles). (E) Raw quantification data and calculated concentration of each Rickettsia strains are presented. (TIF) [file pntd.0003473.s001.tif]
